# Engineered *Escherichia coli* Nissle 1917 with urate oxidase and an oxygen-recycling system for hyperuricemia treatment

**DOI:** 10.1080/19490976.2022.2070391

**Published:** 2022-05-01

**Authors:** Rui Zhao, Zimai Li, Yuqing Sun, Wei Ge, Mingyu Wang, Huaiwei Liu, Luying Xun, Yongzhen Xia

**Affiliations:** a State Key Laboratory of Microbial Technology, Shandong University, Qingdao, Shandong Province, China; b Clinical Laboratory, Qingdao Fuwai Cardiovascular Hospital, Qingdao, Shandong Province, China; c School of Molecular Biosciences, Washington State University, Pullman, WA, USA

**Keywords:** uric acid, hyperuricemia, *Escherichia coli* nissle 1917, urate oxidase, catalase, hemoglobin

## Abstract

Hyperuricemia is the second most prevalent metabolic disease to human health after diabetes. Only a few clinical drugs are available, and most of them have serious side effects. The human body does not have urate oxidase, and uric acid is secreted via the kidney or the intestine. Reduction through kidney secretion is often the cause of hyperuricemia. We hypothesized that the intestine secretion could be enhanced when a recombinant urate-degrading bacterium was introduced into the gut. We engineered an *Escherichia coli* Nissle 1917 strain with a plasmid containing a gene cassette that encoded two proteins PucL and PucM for urate metabolism from *Bacillus subtilis*, the urate importer YgfU and catalase KatG from *E. coli*, and the bacterial hemoglobin Vhb from *Vitreoscilla* sp. The recombinant *E. coli* strain effectively degraded uric acid under hypoxic conditions. A new method to induce hyperuricemia in mice was developed by intravenously injecting uric acid. The engineered *Escherichia coli* strain significantly lowered the serum uric acid when introduced into the gut or directly injected into the blood vessel. The results support the use of urate-degrading bacteria in the gut to treat hyperuricemia. Direct injecting bacteria into blood vessels to treat metabolic diseases is proof of concept, and it has been tried to treat solid tumors.

## Introduction

Elevated uric acid (UA) in human bodies leads to hyperuricemia, which can induce gout, chronic kidney diseases, cardiovascular diseases, and diabetes.^1^ Normal serum UA level in humans is between 178 μM and 416 μM.^2^ UA is produced from purine metabolism, and it cannot be further degraded because two nonsense mutations inactivate the gene encoding urate oxidase (UOX) (EC 1.7.3.3) in humans.^3^ With the risen consumption of high-purine diets, such as meat and shellfish, the incidences of hyperuricemia are gradually increasing in many countries,^4,5^ and hyperuricemia has become the second-largest metabolic disease after diabetes.

Hyperuricemia could be treated either by reducing UA production or promoting UA excretion through the kidney, which are two major factors leading to the development of hyperuricemia.^6^ There are four clinical treatment options. First, diet control is used to reduce the intake of purines and nucleosides.^7^ Second, xanthine oxidase inhibitors such as allopurinol, oxypurinol, and febuxostat, are used to inhibit the activity of xanthine oxidase to reduce UA production.^8^ Third, URAT1 (human urate transporter 1), which is a key UA transporter and is responsible for reabsorbing UA from the renal tubule into cells,^9,10^ is inhibited by drugs such as probenecid, benzbromarone, and losartan to reduce UA absorption in the renal tubules.^11^ Fourth, UA is degraded in the blood by supplementing exogenous UOX.^12^

These clinic therapies have limitations. Restricting diet can significantly reduce the quality of life and the mood of patients, and it must be adhered to for a long time to achieve the therapeutic effect. The use of medicines often has strong side effects, which limits their clinical use.^13,14^ The addition of UOX can significantly reduce the level of UA in the patient’s body in the short term, but studies have also shown that UOX is disruptive in the body by producing H_2_O_2_.^15^ In addition, long-term uses will cause the body to produce antibodies against UOX.^15^

New strategies have been developed to treat hyperuricemia. Several natural products have been successfully tested as xanthine oxidase inhibitors to inhibit UA production or to reduce UA reabsorption in a mouse model.^16^ Exogenous UOX in various forms, such as being modified by polyethylene glycol, encapsulated with catalase or catalase mimic platinum nanoparticles, has successfully applied to reduce UA in mouse blood.^17–19^ These designs are aimed to protect UOX and remove toxic H_2_O_2_.

One-third of the produced UA in humans is excreted through the intestinal tract and is further metabolized by the gut microbiota.^20^ Gut microbiota may be another way to treat hyperuricemia.^21^ Several *Lactobacillus* strains may degrade purine and UA in the gut to ameliorate hyperuricemia in mice.^22,23^ Since UOX activity requires oxygen,^24^ its activity may be reduced in the oxygen-limiting gut.^25,26^ When an engineered *E. coli* BL21 (DE3) strain that overproduces secreted UOX is orally given to rats, it enters the intestine and releases UOX in the gut, resulting in reduced levels of serum UA after five weeks of the treatment.^27^ Yet, the degree of serum reduction is limited. Therefore, an engineered bacterium with enhanced UOX activity under anoxia conditions may be ideal for treating hyperuricemia in the gut.

**Figure 1.**
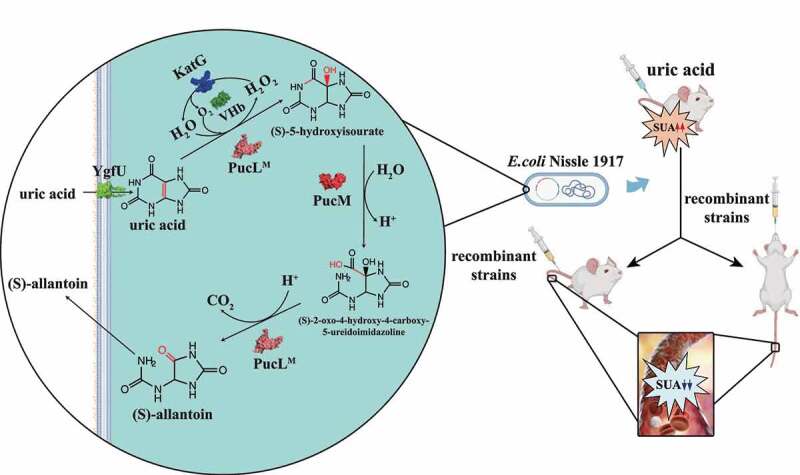
The schematic diagram of an engineered EcN strain for hyperuricemia therapy.EcN was engineered to degrade UA via the pathway in *Bacillus subtilis*. The *ygfU* gene was co-expressed to facilitate UA transport, VHb was used to improve oxygen utilization, and H_2_O_2_, a byproduct of UOX, was eliminated by KatG. The new method to induce hyperuricemia in mice was established by intravenously injecting high concentrated UA. The recombinant strain was used to treat the hyperuricemia mice by oral administration or intravenous injection. Both therapies decreased UA levels of the mice.

UOX is a key enzyme for UA degradation and could be divided into two groups.^24^ The UOX obtained from several microorganisms does not need any cofactor. It has been used as the clinical diagnostic reagent to detect UA and utilized therapeutically to relieve excessive urate accumulation.^28^ Especially, the PucL and PucM from *Bacillus subtilis* are extensively studied. The enzymes catalyze a 3-step UA degradation pathway in *B. subtilis*^29^ (Figure 1). UA is oxidized to 5-hydroxyisourate in step 1, which is catalyzed by the UriC domain of PucL encoding UOX activity. 5-Hydroxyisourate is converted to 2-oxo-4-hydroxy-4-carboxy-5-ureidoimidazoline by hydroxyisourate hydrolase that is encoded by PucM. S-(+)-allantoin is formed by 2-oxo-4-hydroxy-4-carboxy-5-ureidoimidazoline decarboxylase, encoded by the C-terminal domain of PucL. The latter two steps can occur spontaneously,^30^ but step 1 is indispensable.

Several other enzymes may be useful in constructing an engineered bacterium for UA degradation. To overcome oxygen shortage in the gut, the bacterial hemoglobin (VHb) from *Vitreoscilla* sp. is often used.^31^ The *E. coli* catalase KatG efficiently removes H_2_O_2_.^32,33^ Moreover, YgfU from *E. coli* is a proton-gradient-dependent transporter for UA.^34^

Probiotic *E. coli* Nissle 1917 (EcN) has been used to treat inflammatory bowel disease and irritable bowel syndrome for over a century.^35^ Because of its safety record and genetic malleability, it has been engineered to deliver therapeutic payloads to treat several disorders, such as bacterial infection, metabolic disorders, etc.^36^ Recently, it has been developed as a vector to produce and deliver anticancer agents into tumors by intravenous injection.^37^ These applications prove that the injection of EcN into the blood is relatively safe.

We successfully constructed an engineered EcN with PucL, PucM, KatG, Vhb, and YgfU for UA degradation. This strain efficiently degraded UA even with dissolved oxygen (DO) under a 15% level of the normal condition, allowing it to function in the gut. A new method to induce acute hyperuricemia in mice was successfully developed to simulate high UA levels in humans through intravenous injection of UA. The engineered EcN strain was administrated into mice with acute hyperuricemia intragastrically and intravenously, respectively. The serum level of uric acid was sharply reduced by both treatments. Thus, live bacterial therapeutics may have great potentials to treat hyperuricemia.

## Results

### UA degradation pathways engineered in EcN

**Figure 2.**
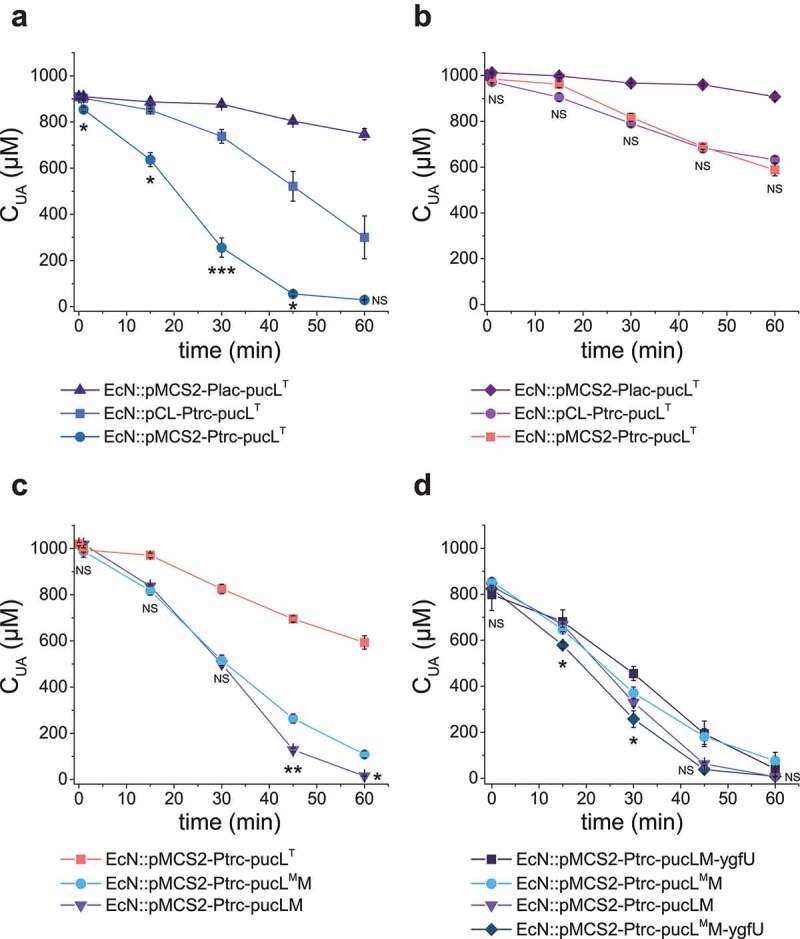
**The optimization of UA degradation by engineering EcN cells.** (a-b). UA degradation by using crude enzymes (a) or whole cells (b) of engineered EcN expressing PucL^T^ in different plasmids under the control of different promoters. (c) UA degradation by EcN whole cells with PucL, PucL^T^, and PucL^M^. (d) UA degradation by EcN whole cells by co-expressing *ygfU*. The degradation curves were determined in HEPES buffer (pH = 7.0) at OD_600_ = 1.0 for whole cells or with proteins at 0.8 mg/mL for enzymatic assays. The UA degradation ability of these whole cells or crude enzyme were assayed at defined time intervals. Three parallel experiments were executed to obtain averages and calculate STDEV. The one-way ANOVA method was used to calculate the *p* value. The Q values were calculated to get the false discovery rate (FDR). Q < 0.05, ‘*’ was marked, Q < 0.01, ‘**’ was marked, Q < 0.001, ‘***’ was marked. In four panels, only the Q value between the mean data of two groups representing the fastest UA degradation rates were shown.

The UriC domain of PucL (PucL^T^) catalyzes the step 1 in the UA degradation (Figure 1), and steps 2 and 3 can occur spontaneously. Two plasmids and two promoters were tested for the expression of a codon-optimized PucL^T^ (Table S1). EcN::pMCS2-Ptrc-pucL^T^ with the gene encoding PucL^T^ under the control of the Ptrc promoter in pBBR1MCS-2 offered the highest activity in the cell extract (Figure 2a); however, the increased activity in cell extracts was not reflected with whole-cell assays (Figure 2b). The cell growth was not affected by the plasmid or promoter used to express *pucL*^T^ (Fig. S1A).

We further compared UA degradation by using EcN::pMCS2-Ptrc-pucL^T^ and EcN::pMCS2-Ptrc-pucLM that overexpressed only PucL^T^ and the whole codon-optimized PucL and PucM, respectively. With cell extracts, the V_max_ of both systems were similar to each other, but the K_m_ of PucLM was greatly decreased (Table 1), suggesting that the cells with the whole pathway can effectively use UA at low concentrations. Indeed, the UA degradation ability of the whole cells was greatly increased (Figure 2c). The activity of PucL^T^ can be improved with D44V and Q268R mutations.^38^ We generated the two site-direct mutations in intact PucL (PucL^M^) and coexpressed it with PucM in EcN (EcN::pMCS2-Ptrc-pucL^M^M). With cell extracts, the V_max_ of PucL^M^M increased ~1.8-fold than that of the PucLM. However, the K_m_ of PucL^M^M increased ~2.0-fold too (Table 1). Due to the increase of Km, the UA degradation ability by the whole cells of EcN::pMCS2-Ptrc-pucL^M^M was also impaired when UA concentrations were low (Figure 2c).

**Table 1.** Kinetic characteristics of crude enzymes in cell extracts of EcN expressing PucL^T^, PucLM, and PucL^M^M

Recombinant EcN	Km (μM)	V_max_ (μmol·min^−1^·mg^−1^)
EcN::pMCS2-Ptrc-pucL^T^	61.03	12.35
EcN::pMCS2-Ptrc-pucLM	17.18	13.88
EcN::pMCS2-Ptrc-pucL^M^M	52.54	38.63

When YgfU, a UA importer in *E. coli*,^34^ was co-expressed with PucL^M^M, the rate of UA degradation rate of EcN::pMCS2-Ptrc-pucL^M^M-ygfU by the whole cells increased (Figure 2d). Unexpectedly, the overexpression of YgfU greatly attenuated the degradation rate of EcN::pMCS2-Ptrc-pucLM-ygfU (Figure 2d). The growth for EcN::pMCS2-Ptrc-pucL^M^M-ygfU was slightly affected, but it could reach a similar cell density as the wild-type strain (Fig. S1B&C). Hence, the fastest UA degradation strain EcN::pMCS2-Ptrc-pucL^M^M-ygfU was used for next step optimization.

### Engineering strain for UA degradation in either hypoxia conditions or anoxia conditions with reduced oxidative stress

**Figure 3.**
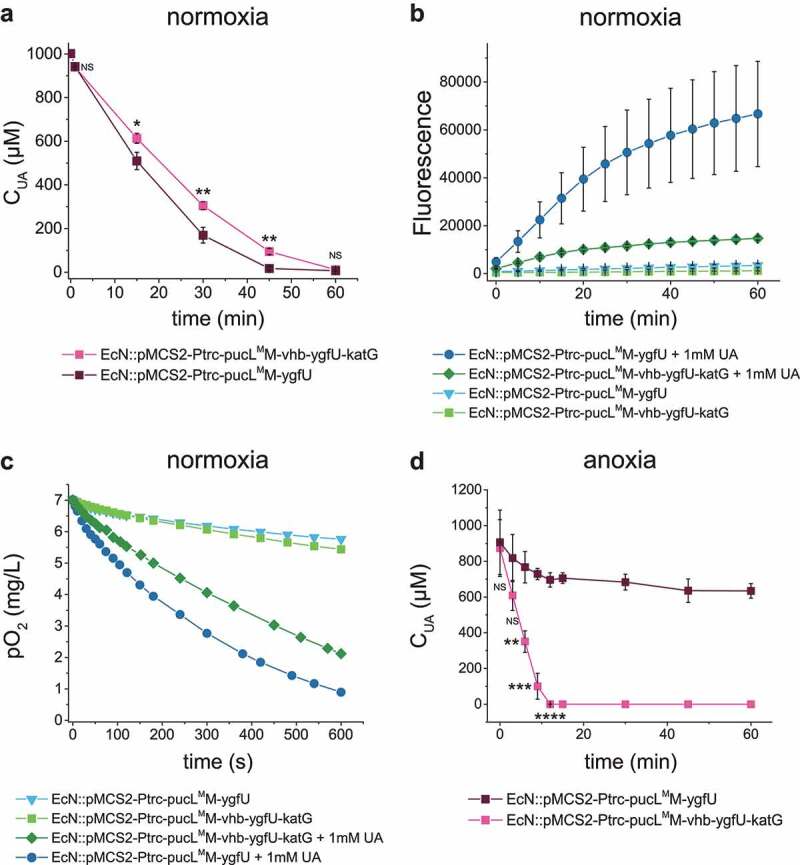
**Vhb and KatG facilitated the recombinant EcN strain for UA degradation under either normal oxygen or hypoxic conditions.** (a) UA degradation by EcN strains under normal oxygen condition. UA degradation under normal oxygen condition was done in flasks with shaking. The ROS level (b) and DO level (c) were also detected. Three parallel experiments were executed to obtain averages and STDEV. (d) UA degradation by EcN strains under hypoxic conditions, where the DO is 15% of the normal oxygen content in medium. UA degradation under hypoxic conditions was done in a bioreactor with controlled DO. The strains were cultured, induced and resuspended into HEPES buffer (50 mM, pH = 7.0) at OD_600_ = 1.0. UA degradation by whole cells were assayed. For the bioreactor experiment, error bars were calculated from the data got in three different batches. The student’s t-test method was used to calculate the *p* value for UA degradation curve. *p* < .05, ‘*’ was marked; *p* < .01, ‘**’ was marked, *p* < .001, ‘***’ was marked, *p* < .0001, ‘****’ was marked.

The *katG* gene encoding *E. coli* catalase was used to convert PucL-generated H_2_O_2_ back to O_2_, and *vhb* encoding a bacterial hemoglobin protein from *Vitreoscilla* sp. C1 was applied to allow the engineered strain to degrade UA at low O_2_ levels. Therefore, EcN::pMCS2-Ptrc-pucL^M^M-vhb-ygfU-katG was constructed to produce PucL^M^M, Vhb, YgfU, and KatG in the same bacterium, which reduced the growth rate, but not the final cell density in LB medium (Fig. S1D). EcN::pMCS2-Ptrc-pucL^M^M-vhb-ygfU-katG degraded UA at a slightly slower rate than EcN::pMCS2-Ptrc-pucL^M^M-ygfU did under normal oxygen condition (Figure 3a), but produced less ROS (Figure 3b). As expected, the presence of KatG and Vhb also recovered the oxygen in this system (Figure 3c). The strain EcN::pMCS2-Ptrc-pucL^M^M-vhb-ygfU-katG could degrade more UA in short time when the DO in medium was restricted around 15% level of normal DO condition (Figure 3d). This modification step facilitated oxygen utilization and relieved possible damage of by-product in the UA degradation process.

### EcN::pMCS2-Ptrc-pucL^M^M-vhb-ygfU-katG reduced serum UA in hyperuricemia mice by transplanting in gut

**Figure 4.**
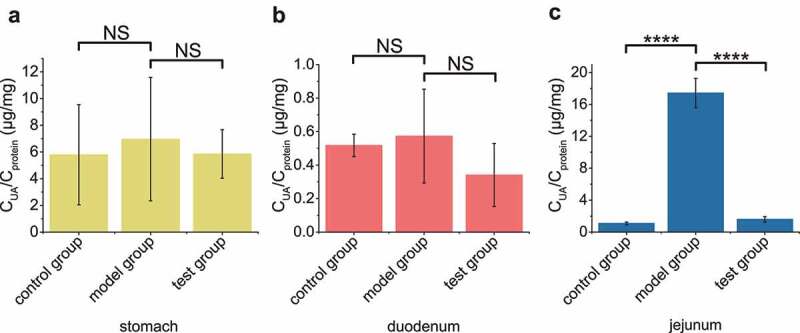
The recombinant EcN strain degraded UA in mouse jejunum. In test group, the optimized engineered EcN strain was oral administered into mice first (n = 6). After 1 hour, the UA was orally administered into these mice. In the positive control group, only UA was orally administered. In the negative control group, neither UA nor EcN was administered. After another hour, the UA levels in stomach (a), duodenum (b) and jejunum (c) were measured. Six parallel experiments were executed to obtain averages and calculate the STDEV. The one-way ANOVA method was used to calculate the *p* value.The Q values were calculated to get the FDR. Q > 0.05, ‘NS’ was marked; Q < 0.0001, ‘****’ was marked.

In order to check if our engineered strain could directly digest UA efficiently in gut, the testing group was intragastrically administered with both UA and the induced bacterial cells, the model group was administered with only UA, and the control group was administered with water. After 30 min, UA concentrations in the stomach and duodenum of the testing group were not significantly different among the three groups (Figure 4a, b), but the UA level in the jejunum of the model group was much higher than those of the testing and control groups (Figure 4c). UA was not found in the ileum (data not shown).

Four reported methods to establish hyperuricemia in mice models were tested. The serum UA reached 2.0-fold higher than the control group only when the 7d-1PO method was used (Fig. S2A), and the UA concentration in gut is not affected (Fig. S2B-D). The induced EcN cells that were administered intragastrically did not ameliorate hyperuricemia (Fig. S3A), nor did it affect the UA concentration in the gut (Fig. S3B-D).

Further inspection revealed that the concentration of serum UA in normal Kunming mice was 14.1 μM ± 6.1 μM (Fig. S2A&S3A), which is 10–24 fold lower than the common range of serum UA concentrations in humans.^39^ UA concentration in the 7d-1PO model was 45.2 μM ± 12.1 μM, still significantly lower than that in humans. At 48 μM UA, the engineered EcN degraded UA at a significantly low rate (Fig. S4A), and potassium oxonate, an inhibitor of UOX, further decreased the UA degradation rate by the engineered EcN cells. Even when potassium oxonate in whole cells was diluted to 10 μM equal to ~ 1% of the amount used for the intraperitoneal administration, it reduced the UA degradation by the EcN cells (Fig. S4B). These two factors could hamper the engineered EcN strain to treat hyperuricemia mice prepared with the 7d-1PO method.

**Figure 5.**
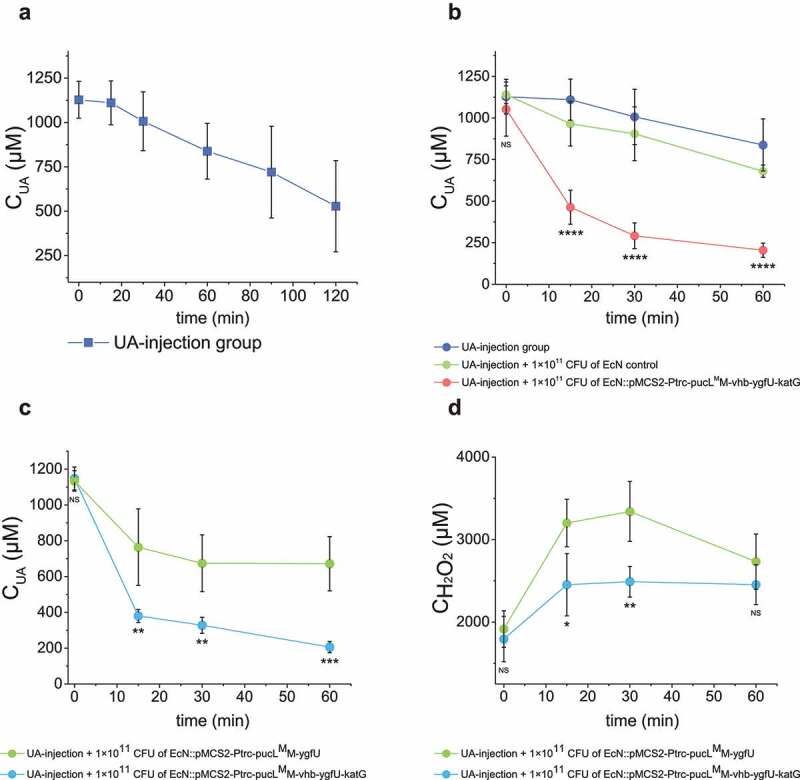
**Therapeutic effect by using the engineered EcN strains via oral administration in the UA-injection hyperuricemia mice.** (a) The serum UA concentrations in mice after the intravenous injection of UA. It is named as the UA-injection group. (b-d) 2 × 10^10^ CFU of the indicated engineered EcN strains containing the UA degrading genes with or without *vhb* and *katG* were orally administered once a day for 5 days in mice. Then UA was intravenously injected 1 hour after the last time of intragastric administration of the EcN strains. The serum UA concentrations were determined (b-c). The serum concentrations of H_2_O_2_ were determined (d). Six parallel experiments were executed to obtain averages and calculate STDEV. In panel b, the one-way ANOVA method was used to calculate the *p* value. The Q values were calculated to get the FDR. Q > 0.05, ‘ns’ was marked; Q < 0.0001, ‘****’ was marked. Only the Q values between the mean data of the two groups representing the fastest UA degradation rates were showed. In panel c&d, the student’s t-test method was used to calculate the *p* value. *p* > .05, ‘ns’ was marked; *p* < .05, ‘*’ was marked; *p* < .01, ‘**’ was marked; *p* < .001, ‘***’ was marked.

To increase serum UA in mice to the levels comparable to that in humans, a UA solution was injected into the blood vessel at 70 mg per kg of body weight (mg/kg) of the testing mice. Serum UA was promoted up to ~1 mM, and the concentration gradually decreased over time (Figure 5a). No mouse was dead due to this treatment. This method was referred to as the UA-injection method. When a UA solution was injected into the blood vessel at 5.7 mg/kg of the testing mice, the serum UA increased to 80 μM at the initial point and was quickly metabolized to normal level in 10 min (Fig. S5A).

EcN::pMCS2-Ptrc-pucL^M^M-vhb-ygfU-katG was used to treat UA-injection hyperuricemia mice. The induced EcN cells were administered by gavage before 70 mg/kg or 5.7 mg/kg of UA was injected intravenously, respectively. In the group injected with 70 mg/kg of UA, the UA level of mice group with oral administration of total 1 × 10^11^ CFU of the EcN strain with the UA degrading genes was decreased sharply compared with the group with gavage of equal amount of EcN cells with the empty vector (Figure 5b). In the group injected with 5.7 mg/kg of UA, the engineered EcN treatment group also could decrease the serum UA level at the initial time point (Fig. S5B). We also tested the effect of VHb and KatG given in the testing mice with EcN::pucL^M^M-vhb-ygfU-katG or EcN::pucL^M^M-ygfU. The overexpression of *vhb* and *katG* in EcN was confirmed to accelerate the rates of UA degradation and H_2_O_2_ removal in the UA-injection hyperuricemia mice (Figure 5c, d). The results indicate that the orally administrated EcN::pucL^M^M-vhb-ygfU-katG cells degrade UA efficiently in the gut and quickly reduce serum UA levels.

### Intravenous administration of EcN::pMCS2-Ptrc-pucL^M^M-vhb-ygfU-katG relieved hyperuricemia in the testing mice

**Figure 6.**
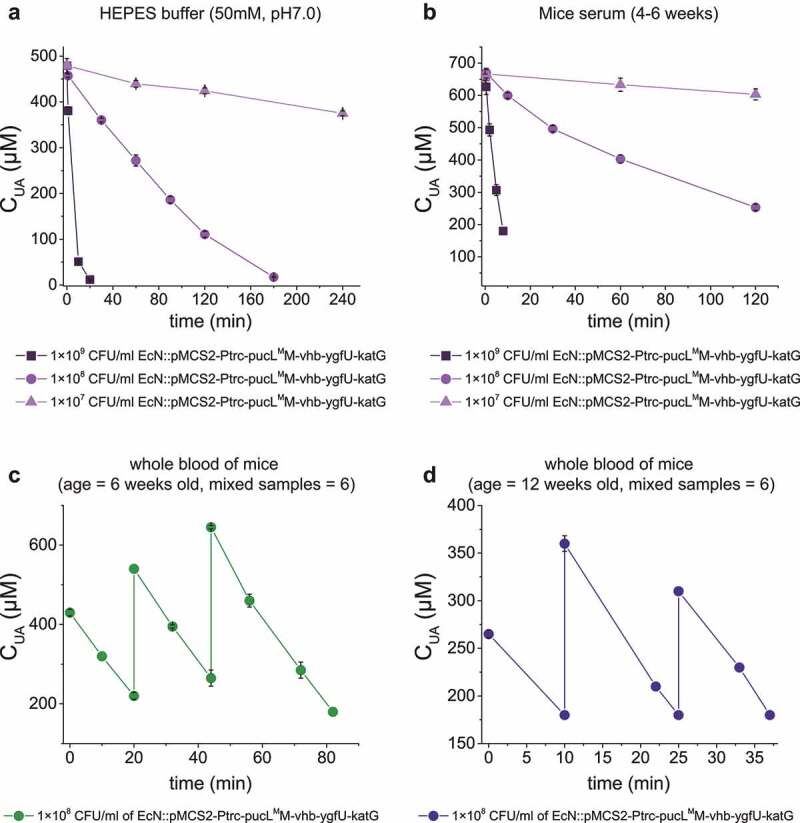
**UA degradation by the engineered EcN strain in buffer, serum and mice blood samples.** (a) UA degradation by the EcN cells in HEPES buffer (50 mM, pH = 7.0). (b) UA degradation by the EcN cells in mice serum. (c) The degradation ability of engineered EcN strain in the mixed blood of young mice (ages at 6 weeks old, mixed blood samples = 6), (d) The degradation ability of engineered EcN strain in the mixed blood of old mice (ages at 12 weeks old, mixed blood samples = 6). The indicated EcN strain was added to degrade UA in the sample, and then UA was added in several rounds. Three parallel experiments were executed to obtain averages and to calculate STDEV.

EcN cells have been intravenously injected to treat tumors. Whether the injection of EcN::pucL^M^M-vhb-ygfU-katG cells into the blood could treat hyperuricemia was tested. First, the induced cells rapidly degraded added UA in HEPES buffer (pH = 7.0) (Control) and commercial mice serum (Figure 6a, b). Second, the whole blood samples of mice were divided into two groups by ages: young group (6 weeks old) and older group (12 weeks old), and a defined concentration of UA was added. When the EcN::pucL^M^M-vhb-ygfU-katG cells (1 x 10^8^ CFU/mL) were added, they rapidly degraded UA in both groups (Figure 6c, d). 320 μM UA was repeatedly added in the whole blood samples, and the UA degradation rates were not altered (Figure 6c, d).

**Figure 7.**
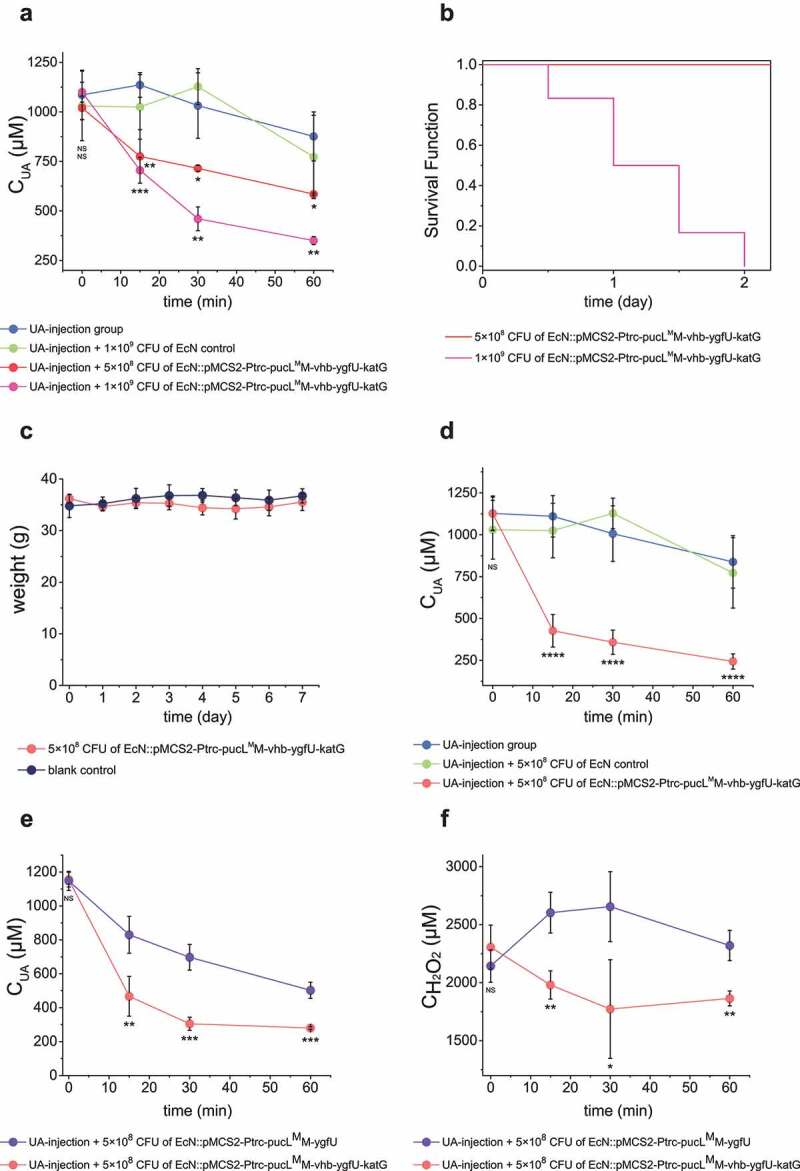
Therapeutic effect by using the engineered EcN strains via intravenous administration in UA-injection hyperuricemia mice. The indicated amount of engineered EcN strains were injected. Then, the UA-injection method was used to induce hyperuricemia in mice. The serum UA levels in different groups were detected at defined time intervals (a, d&e). The serum concentrations of H_2_O_2_ were also determined (f). The time intervals between engineered strain injection and UA injection were either 0 hour (a, e&f) or 10 hours (d). (b) The survival curves of mice in the two groups that were injected with two different amounts of engineered EcN strains were given. (c) Body weight of the mice in the two groups that were either injected with 5 × 10^8^ CFU engineered EcN strain or the same volume of saline (control). Six parallel experiments were executed to obtain averages and calculate the STDEV. For data in panel a&b, the one-way ANOVA method was used to calculate the *p* value. The Q values were calculated to get the FDR. Q < 0.05, ‘*’ was marked; Q < 0.01, ‘**’ was marked; Q < 0.001, ‘***’ was marked; Q < 0.0001, ‘****’ was marked. The Q values represent the comparison between the mean of the indicated group with that of the UA-injection group. For data in panel c&d, the student’s t-test method was used to calculate the *p* value. *p* > .05, ‘ns’ was marked; *p* < .05, ‘*’ was marked; *p* < .01, ‘**’ was marked; *p* < .001, ‘***’ was marked.

Third, 100 μL of the induced cells at 5 × 10^8^ CFU and 1 × 10^9^ CFU were released into the UA-injection hyperuricemia mice at the same time. The serum UA level decreased faster in the treated group than the untreated group (Figure 7a). More engineered EcN offered faster UA degradation (Figure 7a). However, 1 × 10^9^ CFU cells were not suitable for hyperuricemia treatment, since injection of high doses of the EcN cells increased the mortality of mice in 24 hours (Figure 7b). Mice with 100-μL injection of 5 × 10^8^ CFU cells were in good condition, no mouse was died and the weight was not lost even after 7 days of feeding (Figure 7b, c). Fourth, after 5 × 10^8^ CFU EcN cells were injected into the vein of the mice for 8 hours, UA solution was then injected into the vessel at 70 mg/kg to induce hyperuricemia. The UA level decreased much faster in the co-injection group than the control without the EcN cells (Figure 7d). Fifth, when 5.7 mg/kg UA was injected in mice, the engineered EcN strains by intravenous administration also alleviate the increase of UA levels at the start point (Fig. S5B). Sixth, the injection of the EcN strain with *vhb* and *katG* also helped the bacterium to degrade UA and remove H_2_O_2_ faster than the injection of the EcN strain without *vhb* and *katG* (Figure 7e, f).

## Discussion

An engineered EcN strain was constructed for UA degradation. The combination of PucL^M^M and YgfU were necessary, as the modified PucL^M^ increased the enzyme’s catalytic efficiency, as reported,^38^ but decreased its affinity for UA (Table 1). YgfU increases UA uptake and cellular concentrations, which compensates PucL^M^’s low affinity to UA (Figure 2). The DO in animal intestines is limited with a steep oxygen gradient from the serosa to the lumen, and a relatively high level of O_2_ appears in the proximal part of small intestine.^25,26^ Since all known UA degradation pathways require O_2_, Vhb successfully facilitates the bacterium to efficiently use O_2_ at low levels, and KatG detoxifies H_2_O_2_. The engineered EcN strain containing these genes effectively degrades UA under hypoxic conditions (Figure 3d).

We developed a new method to induce hyperuricemia in mice by directly intravenous injection of UA, in which the serum UA concentration is closer to that in human serum (Figure 5a). The UA concentration in the mouse serum is usually low because mice have a functional UOX,^40,41^ and 13.5 ± 1.4 μM is a reported value.^41^ The value we detected for Kunming mice is similar (Fig. S2A&S3A). The engineered EcN strain cannot degrade UA at a meaningful rate at this low level of UA, as the UA degradation rate decreased more than 10 times when the UA concentration was changed from 650 μM to 50 μM (Fig. S4A). Potassium oxonate, which is frequently used to construct traditional hyperuricemia mice,^23,42,43^ further inhibited UA degradation by the EcN strain. To overcome the two hurdles, a new method to induce hyperuricemia in mice was developed by intravenously injecting UA without potassium oxonate.

An *uox*-deletion mouse increases the serum UA level by about 12 folds, but the level is still lower than that in the human serum.^44,45^ Because these UOX-deficient mice are associated with low rate of live births and high mortality, they are not used to test our engineered EcN strain. Intravenous injection of UA sharply increased UA content in the mouse serum by more than 100 times without any death. Since the high UA is kept relatively stable in the mouse serum for the first hour (Fig. 5, 7a&B), it is reasonable to speculate that the injection method provides a testing system for remediating hyperuricemia in mice at least in this short period.

The microbiota has long been known to degrade UA in gut, and it is believed that the degradation may lower UA in the serum.^20,46^ Pioneering studies suggest that *Lactobacillus* spp. in the gut can ameliorate hyperuricemia;^21,23^ however, the mechanism is uncertain, as these bacteria often do not have the UA-degrading ability.^22,47–49^ Thus, direct evidence is needed to support the degradation of UA in the gut to treat hyperuricemia. *E. coli* BL21 (DE3) that produces secreted UOX has been introduced to the gut in hyperuricemia rats, the serum UA is decreased to a limited degree after 5 weeks treatment.^27^ However, free UOX is easily damaged,^17^ especially considering that the intestine is a protease-rich environment.^50^ Oxygen limitation further lowers UOX activity. These factors may contribute to the poor efficacy of using secreted UOX in the gut. In our design, UOX and other enzymes are protected from the unamiable gut environment by EcN cells, allowing effective degradation of UA in the gut and sharply lowering serum UA in hyperuricemia mice (Figure 4c& Figure5b). These results strongly support the use of bacteria to degrade UA in the gut for hyperuricemia treatment.

Intravenous injection of live bacteria for tumor treatments has gained attractions,^51–53^ but the injection for treating metabolic disorders has not been reported. Intravenous injection of the engineered EcN strain can ameliorate hyperuricemia in mice (Figure 7a). UOX has been used to treat hyperuricemia in blood vessels, but the byproduct H_2_O_2_ may lead to side effects.^33,54^ Various ways have been used to solve this problem by using a catalytic cascade of UOX and catalase via diverse nanotechnologies.^17–19,55,56^ EcN can be a good carrier for these enzymes. First, the construction of various combinations of enzymes can be easily achieved in *E. coli*.^57^ Second, the injection of EcN into blood vessels has been shown to be safe, as it does not contain virulence genes and fitness factors that contribute to its colonization and survival within animal hosts.^58^ EcN cells have been widely used as an anti-cancer drug carrier for tumor treatments, and they cannot colonize in the blood and organs for more than 3 days except on solid tumors.^59–61^ Its lipopolysaccharide is recognized by the innate immune system for its elimination from the blood.^62^ Before its elimination, the engineered EcN strain continuously degrades UA, as the UA degrading activity is stable for at least 8 hours after its injection (Figure 7d). As a therapeutic agent for metabolic disease, long retention time in body is preferred. Several methods have been used to promote cell loads and to enhance retention time in the mouse blood.^63,64^ Our work simply offers a potential for using engineered EcN cells as a carrier of enzymes for the treatment of metabolic diseases besides tumors.

This engineered UA-degrading EcN has the potential to treat hyperuricemia mainly from the perspective of proof-of-concept. In order to further promote its practicality, additional work is needed. With the help of synthetic biology, we hope to further enhance the strain’s UA degradation rate through promoter engineering,^65^ RBS engineering,^66^ and expression timing.^67,68^ The change of the order of temporal and spatial expression of genes in this artificial pathway could reduce the heterologous overexpression pressure to the cells and improve their ability to colonize the intestine. As an inspired example, Vincent et. al. recently constructed an EcN engineered strain that could sense an anoxic environment to express enzymes for phenylalanine degradation, in order to develop bacterial therapeutics for treating phenylketonuria.^69^ The strategies basing on plasmid-based system and antibiotic selection are still a common method to engineer probiotics, which could facilitate the optimization process.^69–71^ Yet, this approach could increase the risk for drug-resistant gene transfer. Hence, after the optimization is completed, we will adopt new strategies to keep the cassette from being lost. For instance, we can insert this artificial gene clusters into the bacterial genome.

In summary, a recombinant EcN strain was constructed to efficiently degrade UA under both normal oxygen and hypoxic conditions. The bacterium degrades UA in the gut and blood of mice that was induced with hyperuricemia by intravenously injecting UA. The finding supports the application of bacteria in the gut to degrade UA for hyperuricemia treatment. Besides recombinant bacteria, indigenous gut bacteria that degrade UA may be isolated and used for the treatment. Applying EcN directly in the blood is a new idea to treat metabolic disorders, but more optimizations and efforts are required to achieve the goal.

## Methods and materials

### Strains, plasmids, culture conditions and reagents

The strains and plasmids used in this study are listed in **Table S1**. All the primers are listed in **Table S2**. *E. coli* XL1-Blue MRF’ was used for plasmid construction, and *E. coli* Nissle1917 was used to construct engineered strain for UA degradation. They were cultured at 37°C in Lysogeny broth (LB medium)(#10855001, Oxoid). Kanamycin (A506636, Sangon Biotech), spectinomycin (A600901, Sangon Biotech), and ampicillin (A610028, Sangon Biotech) were used at 50 μg/mL, 50 μg/mL, and 100 μg/mL, respectively.

### DNA manipulations

The *pucL* (Gene ID: 936669) *and pucM* (Gene ID: 937977) from *Bacillus subtilis* were codon-optimized and synthesized by Beijing Genomics Institute (BGI, Beijing). The 975 bp of *pucL* encoding the UriC domain was amplified to obtain truncated *pucL* (*pucL^T^*). Asp-44 and Gln-268 of PucL were mutated to Val and Arg, respectively, in *pucL* and *pucL^T^* by using a modified QuikChange site-directed mutagenesis method.^72^ The mutated *pucL* and *pucL^T^* were named as *pucL^M^* and *pucL^TM^*. The *ygfU* (Gene ID: 949017) and the *katG* (Gene ID: 948431) genes were PCR amplified from *E. coli* MG1655 genomic DNA. The *vgb* gene (Protein ID: WP_019959060.1) from *Vitreoscilla* sp. C1 was codon optimized and synthesized by Beijing Genomics Institute (BGI, Beijing).

Primers used to amplify DNA fragments are given in Table S2. The TEDA method was used for routine plasmid constructions.^73^ The schemes of the constructed plasmids were given in Fig. S6. The detailed plasmid DNA files in the GenBank format were zipped in Dataset S1. Briefly, the *pucL^T^* was cloned into pBBR1MCS-2 and pCL1920 under the control of the Trc promoter to generate pMCS::Ptrc-pucL^T^ and pCL::Ptrc-pucL^T^. The *pucL^T^* was cloned into pBBR1MCS-2 under the P_lac_ promoter to obtain pBBR1MCS-2::Plac-pucL^T^. Plasmids pBBR1MCS-2 and pCL1920 have medium and low copy numbers in *E. coli*, respectively.^74,75^ The Trc promoter has a higher transcription frequency than the Lac promoter.^76^ The *pucL* or *pucL^M^* and *pucM* genes were cloned into pBBR1MCS-2 under the control of the Trc promoter to produce pMCS2::Ptrc-pucLM and pMCS2::Ptrc-pucL^M^M. The *ygfU* was fused into pMCS2::Ptrc-pucL^M^ and pMCS2::Ptrc-pucL^M^M to make pMCS2::Ptrc-pucL^M^-ygfU and pMCS2::Ptrc-pucL^M^M-ygfU, respectively. Further, the *katG* and *vhb* were inserted into plasmid pMCS::Ptrc-pucL^M^M-ygfU to obtain pMCS::Ptrc-pucL^M^M-vhb-ygfU-katG. After plasmids were prepared in *E. coli* XL1-Blue MRF’, they were electroporated into EcN with Eppendorf Eporator^TM^ (BIO-RAD, Irvine, USA) by using 2.5-kV pulse.

### The growth curves and induction of engineered EcN strains

The recombinant strains were inoculated in 5 mL LB with indicated antibiotics in a 14-mL culture tube at 37°C with shaking (200 rpm) overnight. The overnight cultures were inoculated in 50 mL fresh LB with the initial OD_600_ at 0.05 and incubated at 37°C with shaking (200 rpm). For growth curves, cells were grown for 30 min before 2 mM isopropyl-β-D-thiogalactopyranoside (IPTG) (A100487, Sangon Biotech) was added. 1 mL culture was sampled every hour to check the OD_600_ by using the UV-Vis spectrophotometer (UV-1800, Shimadzu, Kyoto, Japan). For induction, 2 mM IPTG was added once the OD_600_ of the cultures reached ~0.6. The cultures were further cultivated at 30°C for 24 hours. The cells were collected, washed, and resuspended into the same HEPES buffer (50 mM, pH = 7.0). The cells were concentrated at 5 × 10^10^ CFU/mL in the potassium phosphate buffer (100 mM, pH 7.5) with 15% glycerol and stored at −80°C. For use, the cells were diluted into the defined OD_600_ as required. The cell density of OD_600_ = 1.0 was treated as 1 × 10^9^ CFU/mL.^77^ We analyzed 12 samples, and results supported this conversion with the average 8.5 × 10^8^ CFU/mL and the standard deviation of 1.2 × 10^8^ CFU·mL^−1^·OD_600_.^−1^

### UA and (S)-allantoin measurements

Four methods were used to determine the concentration of UA in different cases. 1) UA in enzyme assay mixtures or in resting cell suspensions was determined with a spectrophotometer by directly measuring the absorbance at 293 nm.^78^ 2) When UA and its degradation product (S)-allantoin were determined in complex media, an HPLC method was adopted with minor modifications.^79^ Briefly, a C18 reverse phase HPLC column (ODS-A, 250 × 4.6 × 4.6 mm, YMC) was pre-equilibrated with 100% Solvent A (2.5 mM NH_4_H_2_PO_4_ buffered to pH of 3.5 with phosphoric acid) and 0% Solvent B (5% solvent A and 95% methanol). The column was eluted with the following gradients of Solvent B: 0% from 0 to 3.5 min; 40%–80% from 3.5 to 11.5 min; 80%–100% from 11.5 to 15.3 min; 100%–0% from 15.3 to 18 min; 0% from 18 to 22 min. The flow rate was 1.0 mL/min. UA and allantoin were detected by using a HPLC device (LC-20AT, Shimadzu, Japan) with a diode array detector (SPD-20A, Shimadzu, Japan) at absorbance of 205 nm. 3) When low concentrations of UA (≤180 μM) were determined in blood samples, a UA assay kit (Solarbio, Beijing) was used according to the manual instruction. 4) When UA concentration in blood sample was higher than 180 μM, a UA meter PD-G001-3-P (BeneCheck, Beijing, China) was used, which could be operated with simple procedures and low sample volume (<5 μL).

### The crude enzyme assay for UA degradation

Resuspended EcN cells at OD_600_ = 1.0 were broken through a Pressure-cell Homogenizer SPCH-18 (STANSTED, UK). The enzyme activity assay of crude extracts was assayed by referring to a published method.^38^ Briefly, 3.6 mL reaction buffer (50 mM HEPES, pH = 7.0) containing variable concentrations of UA (U2625, Sigma‐Aldrich) at 0, 10, 20, 40, 60, 80, 100, 120, 150 and 200 μM were prepared, and 400 μL crude extract was added to initiate UA degradation. The mixture was incubated at 37°C for 10 min, and the decrease of UA was detected by the spectrophotometric method. The protein concentrations were detected by using a microvolume spectrophotometer (Kaiao Tech, Beijing, China). Kinetic parameters of crude extracts were analyzed following the classical Michaelis–Menten equation, and data was fitted by using the ORIGIN^R^ 2016 software (OriginLab, Northampton, USA).

### UA degradation by EcN whole cells

When UA degradation was assayed in buffer, 20 mL of resuspended induced cells at defined OD_600_ was directly transferred into a 50 mL centrifuge tube. UA was added at 1 mM to initiate the reaction, and the tube was incubated at 37°C with shaking (200 rpm) for 60 min. If necessary, a gradient of potassium oxonate (156124, Sigma‐Aldrich) was added into the reaction mixture to check its inhibition effect for UA degradation. Samples were taken at 15 min intervals. After centrifugation at 13000 rpm for 3 min, the concentrations of UA in supernatant were determined by the spectrophotometric method.

The UA degradation ability of whole cells was also assayed in serum samples or whole blood samples from mice. Commercial mice serum from mice of 4 to 6 weeks of age (SMA100, YZYBIO) was purchased. 2 mL of the whole blood samples were also collected from 6 weeks old and 12 weeks old mice. The prepared whole cells of EcN were harvested and resuspended in the samples of serum or whole blood. Defined concentration of UA was added to initiate the UA degradation assay. The concentrations of UA at defined time intervals were determined by using either the UA assay kit or the UA meter.

### Determination of oxygen consumption and intracellular reactive oxygen species (ROS) for engineered EcN strain

If necessary, the DO and ROS were determined in the UA degradation process *in vitro*. For *in vitro* test, UA was added at 1 mM to 20 mL of resuspended cells at OD_600_ = 1.0. The DO in the reaction mixture was continuously recorded for 10 min with a Versastar RDO meter (Thermo, US). At defined time intervals, the intracellular ROS was detected by using a ROS assay kit (Beyotime S0033, Shanghai, China) according to the manufacturer’s instruction. Briefly, 1 mL of cell suspension was mixed with the ROS probe DCFH-DA. The mixture was incubated at 37°C for 20 min. Then recombinant strains were washed thoroughly to reduce background interference. Fluorescence was measured by using a Synergy H1 microplate reader (Biotek, USA) with excitation at 488 nm and emission at 525 nm.

### Measuring UA degradation rate under oxygen-limiting conditions

Recombinant cells were cultured and harvested in the same way as preparing whole cells. The whole cells were resuspended in 20 mL Brain Heart Infusion Broth (HB8297-1, Hopebio). Defined volume of cell mixtures was immediately inoculated with 700 mL autoclaved Brain Heart Infusion Broth in a 1.4-L Multifors parallel bioreactor (Infors HT, US) at the initial OD_600_ = 1.0, and 10 mL Fetal Bovine Serum (04–121-1A, Biological Industries) was added in a bioreactor as the supplemental nutrient. Following the addition of 2.0 mM IPTG and 50 μg/mL kanamycin, the cells were grown for an additional hour with its DO equal to 15% of the normal condition. Both the eutrophic Brain Heart Infusion Broth and low oxygen were used to simulate intestinal environment. 1.0 mM UA was added in the bioreactor to start UA metabolism under this oxygen-limiting condition. Samples were taken at defined time intervals. After centrifugation, 10 μL of the supernatant was used to measure UA and allantoin by using the HPLC method.

### Animals

Five weeks old (20–25 g) Kunming mice were purchased from Henan Skbex Biotechnology Company. Mice were housed in room (22 ± 2°C) with 65% humidity and 24-hour light-dark cycle. Mice were fed a standard diet. Before the experiments were executed, mice were habituated for a week. Mice were in good health condition at the beginning of the experiments. Mice were randomly divided into groups (n = 6) for subsequent experiments. All animal experiments followed the National Institutes of Health Guide for the Care and Use of Laboratory Animals (NIH Publications No. 8023, revised 1978.) and were approved by the Animal Ethics Committee of Shandong University.

### UA degradation by the engineered EcN strain in mouse intestine

Eighteen 6-week male Kunming mice were classified into three groups (n = 6). The testing group was administered with 200 μL engineered EcN strain by intragastric administration (5 × 10^10^ CFU/mL). Both the positive control group and negative control group were administered with the same volume of HEPES buffer by intragastric administration. One hour later, mice in the testing group and the positive control group were intragastrically administered with 1 mL of 20 mM UA solution in saline. The mice in negative control group were administered with same volume of saline. After 30 min, mice were anesthetized and sacrificed. The stomach and small intestine segments were collected, and different organs were frozen in liquid nitrogen and stored at −80°C for further analysis.

Small pieces of stomach, duodenum, jejunum and ileum tissues from these three groups were cut, weighed and homogenized in 1 mL ice-cold PBS (Phosphate-buffered saline) (pH = 7.4) by using a homogenizer (Tissueprep TP-24, Gering, Beijing, China), and the lysis mixtures were centrifuged (10 min, 10000 rpm at 4°C). The supernatants were utilized to analyze the UA and protein concentrations. The UA concentration was determined by using the UA assay kit. The protein level was determined by using OD_280_, which is detected by a microvolume spectrophotometer (Kaiao Tech, Beijing, China).

### Hyperuricemia mouse models and the treatment with the engineered EcN strain

The hyperuricemia mouse models were established by four known methods: the 7d-1PO method, the 7d-7PO method, the Ade method, and the UA-PO method, according to publications.^23,42,80,81^ 1) Mice induced by the 7d-1PO method were induced by oral administration of adenine (75 mg/kg) for 7 consecutive days, and potassium oxonate at 250 mg/kg was intraperitoneally injected 1 hour after the last adenine administration to inhibit UOX. 2) Mice induced by the 7d-7PO method was similar to the treatment of the 7d-1PO method, but potassium oxonate (250 mg/kg) was intraperitoneally injected 1 hour after each adenine administration. 3) The Ade method orally feeding mice with only adenine (75 mg/kg) for 15 consecutive days. 4) Mice induced by the UA-PO method orally received UA (290 mg/kg) and potassium oxonate (570 mg/kg) for 15 consecutive days. Adenine, UA, and potassium oxonate were suspended in 0.5% sodium carboxymethyl cellulose (CMC-Na). The control group was orally administered with the same volume of 0.5% CMC-Na.

Mice induced with the 7d-1PO method were selected to receive the treatment with the engineered EcN strain. The mice were divided into three groups, including testing group, model group, and control group. In the model group, the hyperuricemia mice were established according to the 7d-1PO method without treatment with the engineered EcN strain. In the testing group, they were intragastrically administered with 200 μL of the engineered EcN strain (5 × 10^10^ CFU/mL) 8 hours before adenine administration for 7 consecutive days. In the control group, the mice were given the same volume of the buffer (100 mM potassium phosphate, pH 7.5, with 15% glycerol).

Mice were fasted for 20 hours and were anesthetized by ether (10009318, Sinopharm) 1 hour after the final drug administration, and then blood samples were collected via eyeball enucleation. Blood samples were stored at −20°C until UA analysis; small intestine tissues were obtained after mice were sacrificed, frozen in liquid nitrogen, and stored at −80°C until analyses.

### The development of a new method to induce hyperuricemia in mice

Eighteen 6-week male Kunming mice were divided into three groups (n = 6). The two testing groups were tail intravenously injected with a UA solution at 70 mg/kg or 5.7 mg/kg as indicated. The UA injections with the high amount and low amount were used to promote serum UA in a physiologically relevant concentration to humans and mice, respectively. The control group was intravenous injected only with saline. The UA concentration was determined from blood samples taken from mouse tails at defined time intervals. The UA concentrations in these organs were determined by using the UA assay kit (Solarbio, Beijing). The mice received UA injection had high UA in the sera and termed UA-injection hyperuricemia mice.

### The treatment of hyperuricemia mice with the engineered EcN strain

The engineered EcN strain was used to treat the hyperuricemia mice by either intragastric or intravenous administration. 1) For intragastric treatment, 18 6-week male Kunming mice were divided into three groups: the two testing groups and the control group. Defined amount of the engineered EcN cells and EcN cells with the empty vector were administered into mice in the two testing groups intragastrically. The mice in the control group was injected with the same amount of buffer. All mice in the three groups were intravenously injected with the UA solution at 70 mg/kg. Mice in the intragastric administration group were orally administrated with 2 × 10^10^ CFU of the EcN cells of two strains for 5 consecutive days, UA solution was intravenously injected 1 hour after the last bacterial administration at day 5. 2) For intravenous treatment, 24 6-week male Kunming mice were divided into four groups: three testing groups and a control group. Defined amount of EcN cells with pMCS2-Ptrc-pucL^M^M-vhb-ygfU-katG, pMCS2-Ptrc-pucL^M^M- ygfU, or the empty vector pMCS2 were administered into mice in the three testing groups. The mice in the control group was injected with the same amount of buffer instead. The mice in the intravenous administration of EcN received the bacterial injection first, and then the UA solution was injected into the mice either immediately or 10 hours after the bacterial injection.

For both treatments, the UA concentration was determined with UA meter from blood samples taken from mouse tail at defined time intervals. For intravenous treatment, the weight and survival numbers of the mice in each groups were recorded at defined time intervals after EcN was injected to draw survival curves and weight curves.

### The determination of H_2_O_2_ production in vivo

The H_2_O_2_ concentration was determined during UA degradation *in vivo*. The engineered EcN strains with oxygen-recycling genes or not were used to treat the hyperuricemia mice by either intragastric or intravenous administration, as described above. After the blood samples were taken, the H_2_O_2_ levels were detected by using a H_2_O_2_ assay kit (Beyotime S0038, Shanghai, China) according to the manufacturer’s instruction. Meanwhile, the UA concentration was determined with the UA meter.

### Statistical analysis

Statistical analysis was done using GraphPad Prism 9.0. Data in more than two groups were analyzed using independent one-way analysis of variance (ANOVA) to calculate the *p* values of indicated pairs of group. Q values were calculated according to the Benjaminiad Hochberg method to represent the adjusted *p* value. Q < 0.05 indicated statistical significance. The significant differences between two groups were analyzed using an independent student’s t-test. The *p* value < .05 indicated statistical significance. Further, the relevant data were fitted by using the ORIGIN^R^ 2016 software (OriginLab, Northampton, USA).

## Supplementary Material

Supplemental Material

## Data Availability

The authors confirm that the data supporting the findings of this study are available within the article and its supplementary materials.
