# Supplementary material for: Engineered Escherichia coli Nissle 1917 with urate oxidase and an oxygen-recycling system for hyperuricemia treatment
Source: Gut Microbes. 2022 May 1;14(1):2070391. doi: 10.1080/19490976.2022.2070391 (PMC9067508; doi:10.1080/19490976.2022.2070391)
Supplement: Supplemental Material [file KGMI_A_2070391_SM8590.zip › 11/Supplementary file - figures & tables.docx]

**Supplementary information for**

**Engineered *Escherichia coli* Nissle 1917 with Urate Oxidase and an Oxygen-recycling System for Hyperuricaemia Treatment**

Rui Zhao^1^, Zimai Li^1^, Yuqing Sun^1^, Wei Ge^3^, Mingyu Wang^1^, Huaiwei Liu^1^, Luying Xun^1,2^, Yongzhen Xia^1*^

*^1^State Key Laboratory of Microbial Technology, Shandong University, Qingdao, 266237, People’s Republic of China*

*^2^School of Molecular Biosciences, Washington State University, Pullman, WA, 99164-7520, USA*

*^3^Clinical laboratory, Qingdao Fuwai Cardiovascular Hospital, Qingdao 266034, People’s Republic of China*

* To whom correspondence should be addressed to Y. Xia. Tel: +86-532-58631572; Email: [xiayongzhen2002@sdu.edu.cn](mailto:xiayongzhen2002@sdu.edu.cn)

Including

Table S1 - Table S2

Figure S1 - Figure S6

**Supplementary Table S1. Strains and plasmids used in this study.**

| **Strains or plasmids** | **Features** | **Origin** |
| --- | --- | --- |
| **Strains** | | |
| *E. coli* XL1-Blue MRF’ | ∆(mcrA)183∆(mcrCB-hsdSMR-mrr)173 endA1 supE44 thi-1 recA1 gyrA96 relA1 lac [F ́ proAB  lacIqZ∆M15Tn10 (Tet^r^)] | Stratagene |
| *E.coli* str. K-12 substr. MG1655 | Wild type | Our lab |
| *E. coli* Nissle 1917 | Wild type | Our lab |
| EcN::pCL-Ptrc-pucL^T^ | *E. coli* Nissle 1917 with pCL1920-Ptrc-pucL^T^ | This study |
| EcN::pMCS2-Plac-pucL^T^ | *E. coli* Nissle 1917 with pBBR1MCS2-Plac-pucL^T^ | This study |
| EcN::pMCS2-Ptrc-pucL^T^ | *E. coli* Nissle 1917 with pBBR1MCS2-Ptrc-pucL^T^ | This study |
| EcN::pMCS2-Ptrc-pucLM | *E. coli* Nissle 1917 with pBBR1MCS2-Ptrc-pucLM | This study |
| EcN::pMCS2-Ptrc-pucL^M^M | *E. coli* Nissle 1917 with pBBR1MCS2-Ptrc-pucL^M^M | This study |
| EcN::pMCS2-Ptrc-pucL^T^-ygfU | *E. coli* Nissle 1917 with pBBR1MCS2-Ptrc-pucL^T^-ygfU | This study |
| EcN::pMCS2-Ptrc-pucLM-ygfU | *E. coli* Nissle 1917 with pBBR1MCS2-Ptrc-pucLM-ygfU | This study |
| EcN::pMCS2-Ptrc-pucL^M^M-ygfU | *E. coli* Nissle 1917 with pBBR1MCS2-Ptrc-pucL^M^M-ygfU | This study |
| EcN::pMCS2-Ptrc-pucL^M^M-vhb-ygfU-katG | *E. coli* Nissle 1917 with pBBR1MCS2-Ptrc- pucL^M^M-vhb-ygfU-katG | This study |
| **Plasmids** | | |
| pBBR1MCS-2 | Kan^R^, pBBR ori | ^1^ |
| pCL1920 | Spc^R^, pSC101 ori | ^2^ |
| pTrc99a | Amp^R^, pBR322 ori, Trc promoter | ^3^ |
| pBBR1MCS-2::Plac-pucL^T^ | Derived from pBBR1MCS-2, *pucL^T^* gene was under the control of *lac* promoter | This study |
| pCL1920::Ptrc-pucL^T^ | Derived from pCL1920, *pucL^T^* gene was under the control of *trc* promoter | This study |
| pBBR1MCS-2::Ptrc-pucL^T^ | Derived from pBBR1MCS-2, *pucL^T^* gene was under the control of *trc* promoter | This study |
| pBBR1MCS-2::Ptrc-pucLM | Derived from pBBR1MCS-2, *pucL* and *pucM* genes were under the control of trc promoter | This study |
| pBBR1MCS-2::Ptrc-pucL^M^M | Derived from pBBR1MCS-2, *pucL^M^* and *pucM* genes were under the control of trc promoter | This study |
| pBBR1MCS-2::Ptrc-pucL^T^-ygfU | Derived from pBBR1MCS-2, *pucL^T^* and *ygfU* genes were under the control of trc promoter | This study |
| pBBR1MCS-2::Ptrc-pucLM-ygfU | Derived from pBBR1MCS-2, *pucL*, *pucM* and *ygfU* genes were under the control of trc promoter | This study |
| pBBR1MCS-2::Ptrc-pucL^M^M-ygfU | Derived from pBBR1MCS-2, *pucL^M^*, *pucM* and *ygfU* genes were under the control of trc promoter | This study |
| pBBR1MCS-2::Ptrc-pucL^M^M-vhb-ygfU | Derived from pBBR1MCS-2, *pucL^M^*, *pucM*, *vhb* and *ygfU* genes were under the control of trc promoter | This study |
| pBBR1MCS-2::Ptrc-pucL^M^M-vhb-ygfU-katG | Derived from pBBR1MCS-2, *pucL^M^*, *pucM*, *vhb*, *ygfU* and *katG* genes were under the control of trc promoter | This study |

**Supplementary Table S2. The synthetic oligos used in this study.**

| Primers name | Oligo sequences | Purpose |
| --- | --- | --- |
| pCL1920-backbone-fr | AATTCACTGGCCGTCGTTTTAC | To amplify the backbone of pCL1920 from pCL1920 and used for pCL1920::Ptrc-pucL^T^ cloning |
| pCL1920-backbone-rev | AATTCCCGACAGTAAGACGGG |  |
| LacI-ptrc-pCL1920-fr | CCGTCTTACTGTCGGGAATTGACACCATCGAATGGTGCAA | To amplify the lacI gene and Ptrc promoter fragment of pTrc99a from pTrc99a and used for pCL1920::Ptrc-pucL^T^ cloning |
| LacI-ptrc-pCL1920-rev | GTGTGAAATTGTTATCCGCTCACAATTCC |  |
| PucL^T^-1920-fr | AGCGGATAACAATTTCACACTTTAACTTTAAGGAGGAGATATACCATGAAACGCACCATGAGCTATGG | To amplify the pucL^T^ gene from synthesized pucL gene and used for pCL1920::Ptrc-pucL^T^ cloning |
| PucL^T^-1920-rev | AAAACGACGGCCAGTGAATTTTACGCTTTCAGGCTGCGGC |  |
| pBBRMCS2-backbone-fr | GTTAAAATTCGCGTTAAATTTTTGTTAAATCAGC | To amplify the backbone of p BBRMCS-2 from pBBRMCS-2 and used for pBBRMCS-2::Ptrc-pucL^T^ cloning |
| pBBRMCS2-backbone-rev | GGCGCATGCATAAAAACTGT |  |
| LacI-ptrc-PucL^T^-MCS2-fr | ACAGTTTTTATGCATGCGCCGACACCATCGAATGGTGCAAAACC | To amplify the lacI gene, Ptrc promoter and pucL^T^ gene from pCL1920::Ptrc-pucL^T^ and used for pBBRMCS-2::Ptrc-pucL^T^ cloning |
| LacI-ptrc-PucLT-MCS2-rev | AATTTAACGCGAATTTTAACTTACGCTTTCAGGCTGCGGC |  |
| MCS2_plac_PucL^T^_fr | AGGCACCCCAGGCTTTACACTTTATGCTTCCGGCTCGTATGTTGTGTGGAATTGTGAGCGGATAACAATTTCACACTTTAACTTTAAGGAG | To amplify backbone of pBBRMCS-2::Ptrc-pucL^T^ and replace ptrc promoter to plac promoter, and used for pBBRMCS-2::Plac-pucLT constructing. |
| MCS2_plac_PucL^T^_rev | AGTGTAAAGCCTGGGGTGCCTAATGAGTGAGCTAACTCACATTACAGCTCATTTCAGAATATTTGCCAGAACCGTTATGATGTCG |  |
| pucL^TM^_MCS2_fr | CTTTAAGGAGGAGATATACCATGAAACGCACCATGAGCTATG | To amplify the pucL^TM^ gene from synthesized pucL^M^ gene and used for pBBRMCS-2::Ptrc-pucL^TM^ cloning |
| pucL^TM^_MCS2_rev | AATTTAACGCGAATTTTAACTTACGCTTTCAGGCTGCG |  |
| pBBRMCS2-lacI-ptrc-backbone-fr | GTTAAAATTCGCGTTAAATTTTTGTTAAATCAGC | To amplify the backbone of p BBRMCS-2, lacI gene and ptrc promoter from pBBRMCS-2::Ptrc-pucL^T^ and used for pBBRMCS-2::Ptrc-pucL^TM^, pBBRMCS-2::Ptrc-pucLM and pBBRMCS-2::Ptrc-pucL^M^M cloning |
| pBBRMCS2-lacI-ptrc-backbone-rev | GGTATATCTCCTCCTTAAAGTTAAAGTGTG |  |
| pucLM_MCS2_fr | CTTTAAGGAGGAGATATACCATGTTTACCATGGATGATCTGAACCAGATG | To amplify the pucL or pucL^M^ gene and pucM gene from synthesized pucL or pucL^M^ gene and pucM gene, and used for pBBRMCS-2::Ptrc-pucLM and pBBRMCS-2::Ptrc-pucL^M^M cloning |
| pucLM_MCS2_rev | AATTTAACGCGAATTTTAACTTAGCTGCCGCGATACACCTG |  |
| pBBRMCS2-lacI-ptrc-pucL^TM^-backbone-fr | GTTAAAATTCGCGTTAAATTTTTGTTAAATCAGC | To amplify the backbone of pBBRMCS-2, lacI gene, ptrc promoter and pucL^TM^ gene from pBBRMCS-2::Ptrc-pucL^TM^ and used for pBBRMCS-2::Ptrc-pucL^TM^-ygfU cloning |
| pBBRMCS2-lacI-ptrc-pucL^TM^-backbone-rev | TGGGAATCTATGGCGCTCATCTAGTATTTCCCCTCTTTTTACGCTTTCAGGCTGCGG |  |
| pBBRMCS2-lacI-ptrc-pucLM-backbone-fr | GTTAAAATTCGCGTTAAATTTTTGTTAAATCAGC | To amplify the backbone of pBBRMCS-2, lacI gene, ptrc promoter, pucL or pucL^M^ gene and pucM gene from pBBRMCS-2::Ptrc-pucLM or pBBRMCS-2::Ptrc-pucL^M^M and used for pBBRMCS-2::Ptrc-pucLM-ygfU and pBBRMCS-2::Ptrc-pucL^M^M-ygfU cloning |
| pBBRMCS2-lacI-ptrc-pucLM-backbone-rev | TGGGAATCTATGGCGCTCATCTAGTATTTCCCCTCTTTTTAGCTGCCGCGATACACCTG |  |
| ygfU-MCS2-fr | ATGAGCGCCATAGATTCCCAAC | To amplify the ygfU gene from *E. coli* MG1655 genome, and used for pBBRMCS-2::Ptrc-pucL^TM^-ygfU pBBRMCS-2::Ptrc-pucLM-ygfU and pBBRMCS-2::Ptrc-pucL^M^M-ygfU cloning |
| ygfU-MCS2-rev | AATTTAACGCGAATTTTAACTTATTCTCCATGCTCATTTTTCTTCAGCT |  |
| vhb-MCS2-fr | AGGTGTATCGCGGCAGCtaaTCTAGAGATTAAAGAGGAGAATACTAGATGCTGGATCAGCAGACCATTAACAT | To amplify the vhb gene from synthesized vhb, and used for pBBRMCS-2::Ptrc-pucL^M^M-vhb-ygfU cloning |
| vhb-MCS2-rev | ATCTAGTATTTCCCCTCTTTTTACTCCACTGCCTGTGCATACAG |  |
| katG-MCS2-fr | AAAATGAGCATGGAGAATAACTAGAGAAAGAGGAGAAATACTAGATGAGCACGTCAGACGATATCCATAACACC | To amplify the katG gene from *E. coli* MG1655 genome , and used for pBBRMCS-2::Ptrc-pucL^M^M-vhb-ygfU-katG cloning |
| katG-MCS2-rev | AATTTAACGCGAATTTTAACTTACAGCAGGTCGAAACGGTCGAG |  |
| pBBRMCS2-lacI-ptrc-pucL^M^M-ygfU-backbone-fr | AAAGAGGGGAAATACTAGATGAGCGCC | To amplify the backbone of pBBRMCS-2, lacI gene, ptrc promoter, pucL^M^ gene, pucM gene and ygfU gene from pBBRMCS-2::Ptrc-pucL^M^M-ygfU and used for pBBRMCS-2::Ptrc-pucL^M^M-vhb-ygfU cloning |
| pBBRMCS2-lacI-ptrc-pucL^M^M-ygfU-backbone-rev | TTAGCTGCCGCGATACACCTG |  |
| pBBRMCS2-lacI-ptrc-pucL^M^M-vhb-ygfU-backbone-fr | GTTAAAATTCGCGTTAAATTTTTGTTAAATCAGC | To amplify the backbone of pBBRMCS-2, lacI gene, ptrc promoter, pucL^M^ gene, pucM gene, vhb gene and ygfU gene from pBBRMCS-2::Ptrc-pucL^M^M-vhb-ygfU and used for pBBRMCS-2::Ptrc-pucL^M^M-vhb-ygfU-katG cloning |
| pBBRMCS2-lacI-ptrc-pucL^M^M-vhb-ygfU-backbone-rev | TTATTCTCCATGCTCATTTTTCTTCAGC |  |


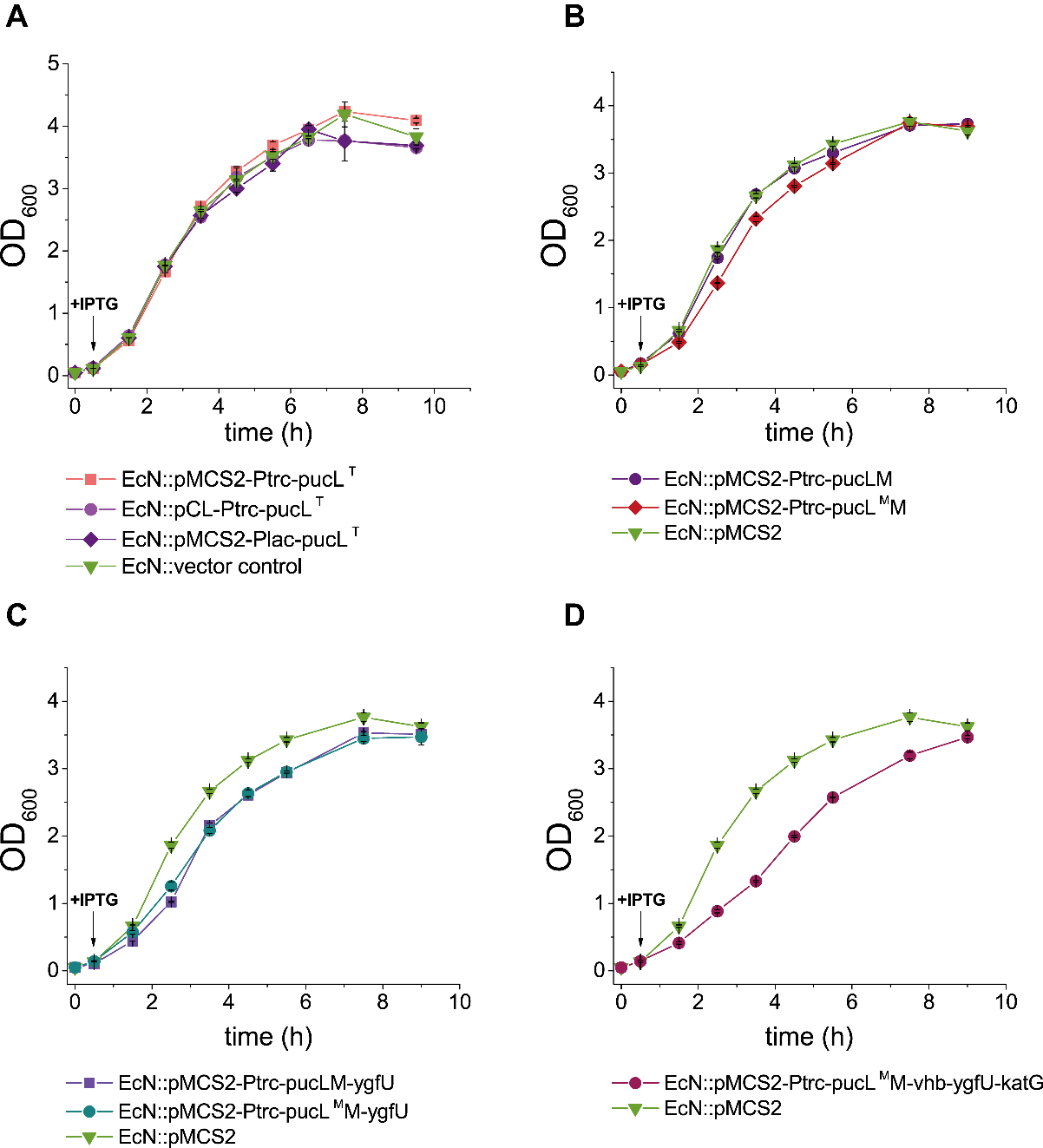


**Supplementary Figure S1. Growth curves of recombinant strains.** The bacteria were cultured in LB medium overnight and then transferred into fresh LB medium. IPTG was added to 1 mM after the cultures were incubated at 37 ^o^C for 30 min. The OD_600_ was detected at defined time intervals. Three parallel experiments were executed to calculate the STDEV.


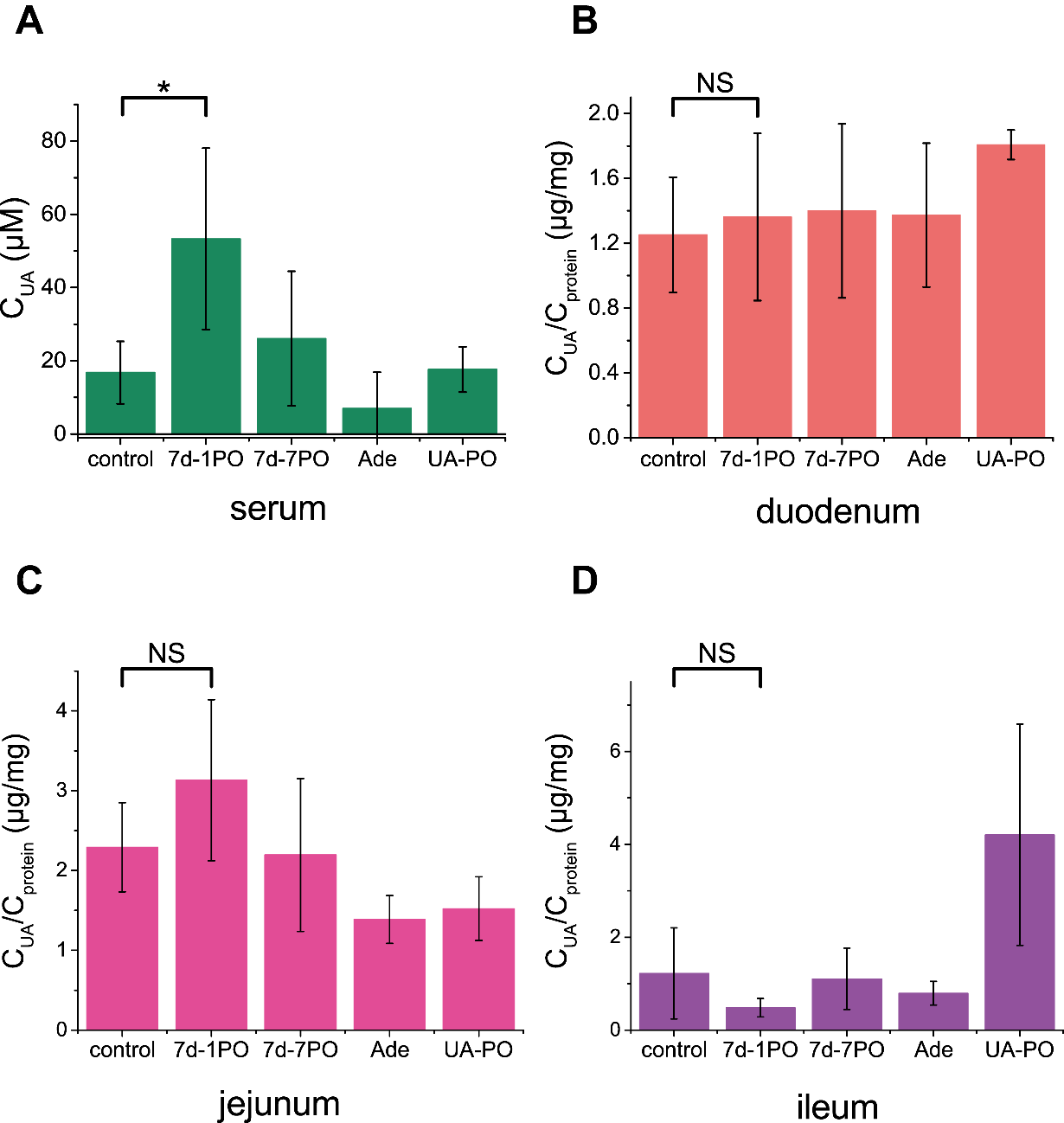


**Supplementary Figure S2.** **The UA levels of mice in serum and gut from HUA mice prepared with four different methods.** Four different methods were used to produce HUA mice. In the control group, no treatment was done. The UA levels of the mice in serum (A), duodenum (B), jejunum (C) and ileum (D) were determined. Six mice were used in one group to calculate STDEV. The one-way ANOVA method was used to calculate the *p* value. The Q values were calculated to get the false discovery rate. Q < 0.05, ‘*’ was marked; Q > 0.05, ‘NS’ was marked.


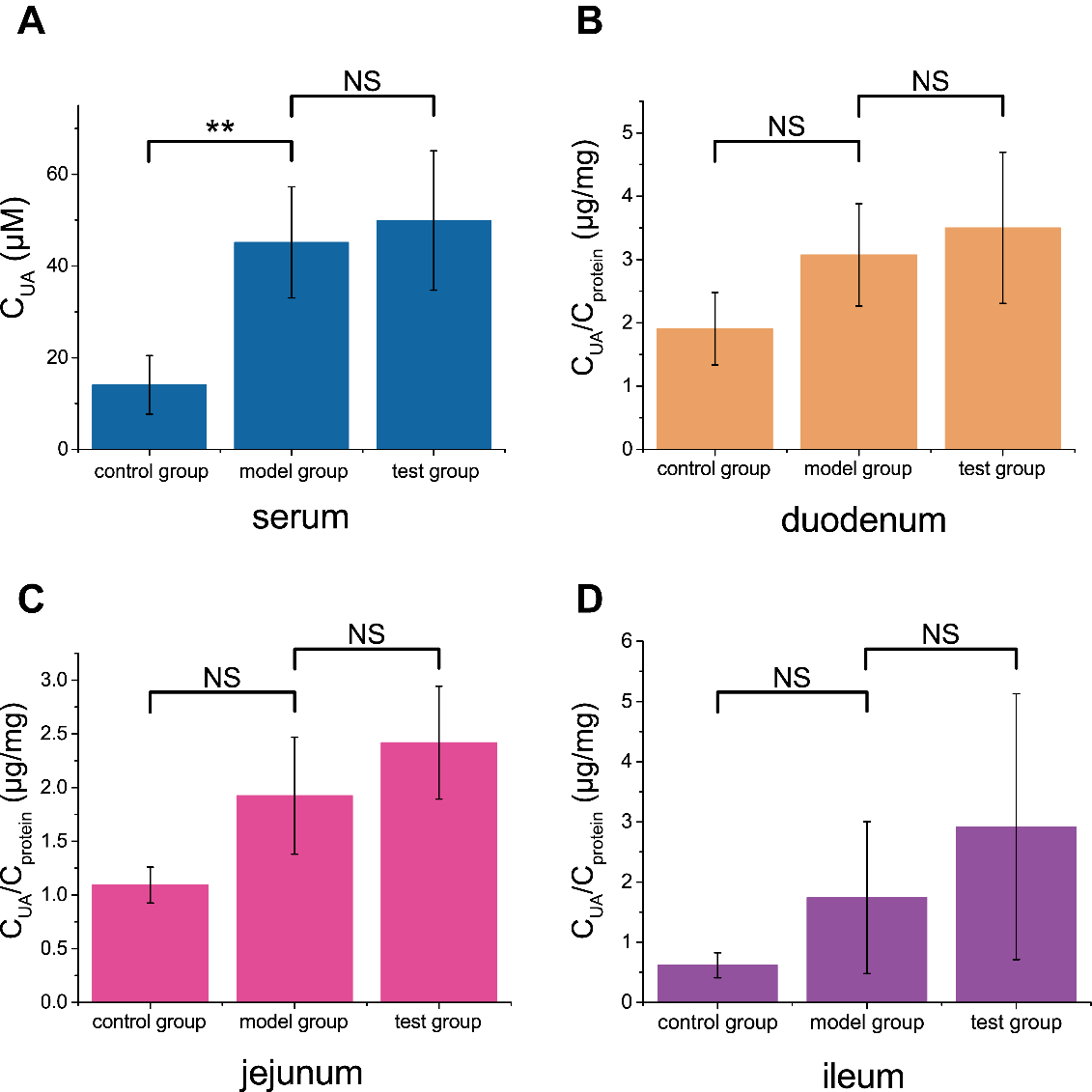


**Supplementary Figure S3.** **UA level in HUA mice prepared with the 7d-1PO method and treated with the engineered EcN strain.** In the control group, no treatment was done. In the model group and test group, the HUA mice were established with the 7d-1PO method. In the test group, the engineered EcN strain was applied to treat HUA. After treatment, the mice in all the three groups were sacrificed, and the UA level of the mice in serum (A), duodenum (B), jejunum (C), and ileum (D) were determined. Six mice were used in each group to calculate STDEV. The one-way ANOVA method was used to calculate the *p* value. The Q values were calculated to get the false discovery rate. Q < 0.01, ‘**’ was marked; Q > 0.05, ‘NS’ was marked.


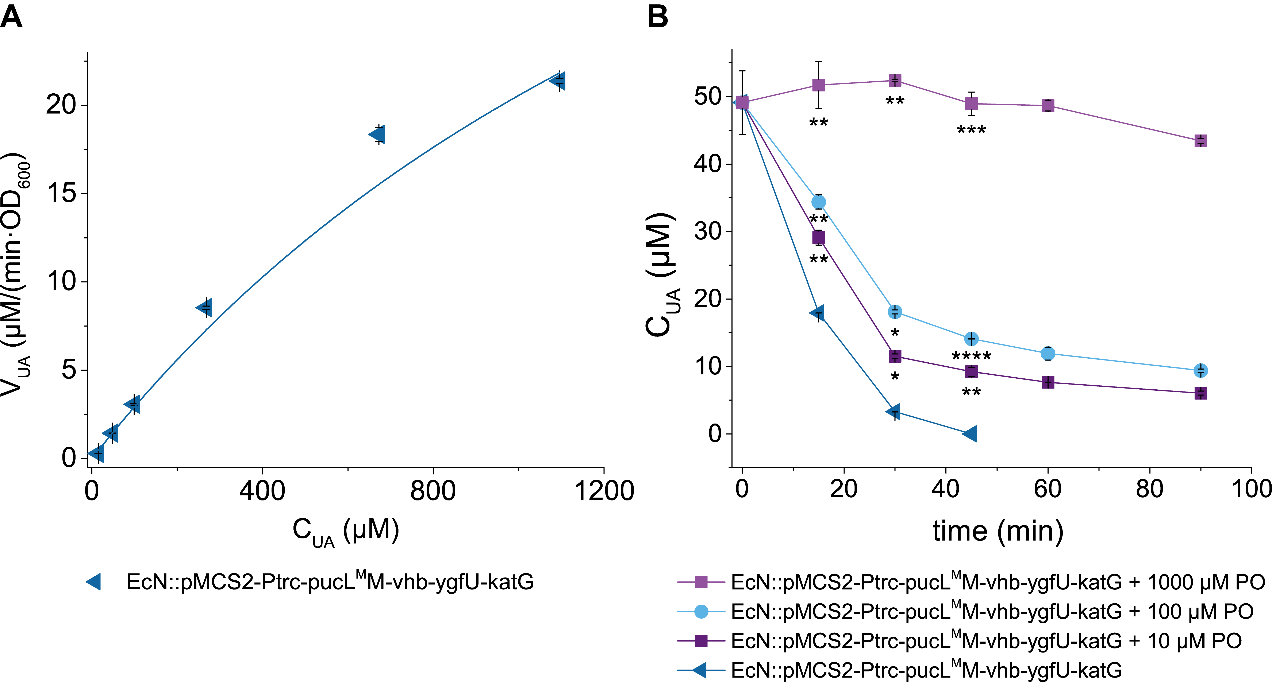


**Supplementary Figure S4.** **Effect of UA concentrations and potassium oxonate on UA degradation by** **the engineered EcN strain.** A) The degradation rate by whole cells at different UA concentrations. B) Poassium oxonate inhibited UA degradation by the engineered EcN cells. Three parallel experiments were executed to calculate STDEV. The one-way ANOVA method was used to calculate the *p* value. The Q values were calculated to get the false discovery rate. Q < 0.05, ‘*’ was marked; Q < 0.01, ‘**’ was marked; Q < 0.001, ‘***’ was marked; Q < 0.0001, ‘****’ was marked. The Q value represents the compare of the mean of three Poassium oxonate treatment groups with the mean of the untreatment group, respectively.
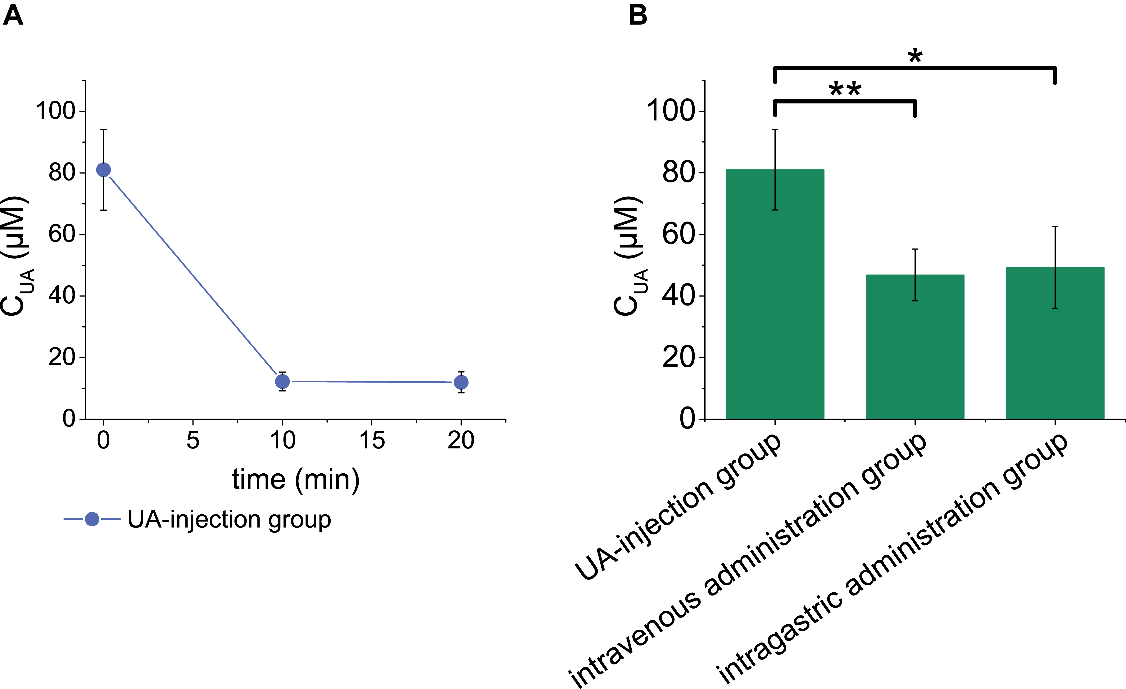


**Supplementary Figure S5. The establishment and treatment for the UA-injection hyperuricaemia mice with physiologically relevant UA concentrations to mice. A**) The serum UA concentrations in mice was taken in defined time intervals and determined after the tail intravenous injection of 5.7 mg/kg UA. **B**) For the UA-injection group, only 5.7 mg/kg UA was injected through tail vein. For the intravenous administration group, the procedures were followed according to intravenous treatment. 100 μL 5×10^9^ CFU/mL engineered EcN::pMCS2-Ptrc-pucL^M^M-vhb-ygfU-katG was used. For the intragastric administration group, the procedures were followed according to intragastric treatment. 200 μL 1×10^11^ CFU/mL engineered EcN::pMCS2-Ptrc-pucL^M^M-vhb-ygfU-katG was used once a day for 5 days. For both treatment groups, the engineered strains were given before UA injection. For all the three groups, the serum UA was determined immediately after UA injection. The operation time for serum taken were kept around 30 s. The one-way ANOVA method was used to calculate the *p* value. The Q values were calculated to get the false discovery rate. Q < 0.05, ‘**’ was marked; Q < 0.01, ‘**’ was marked.


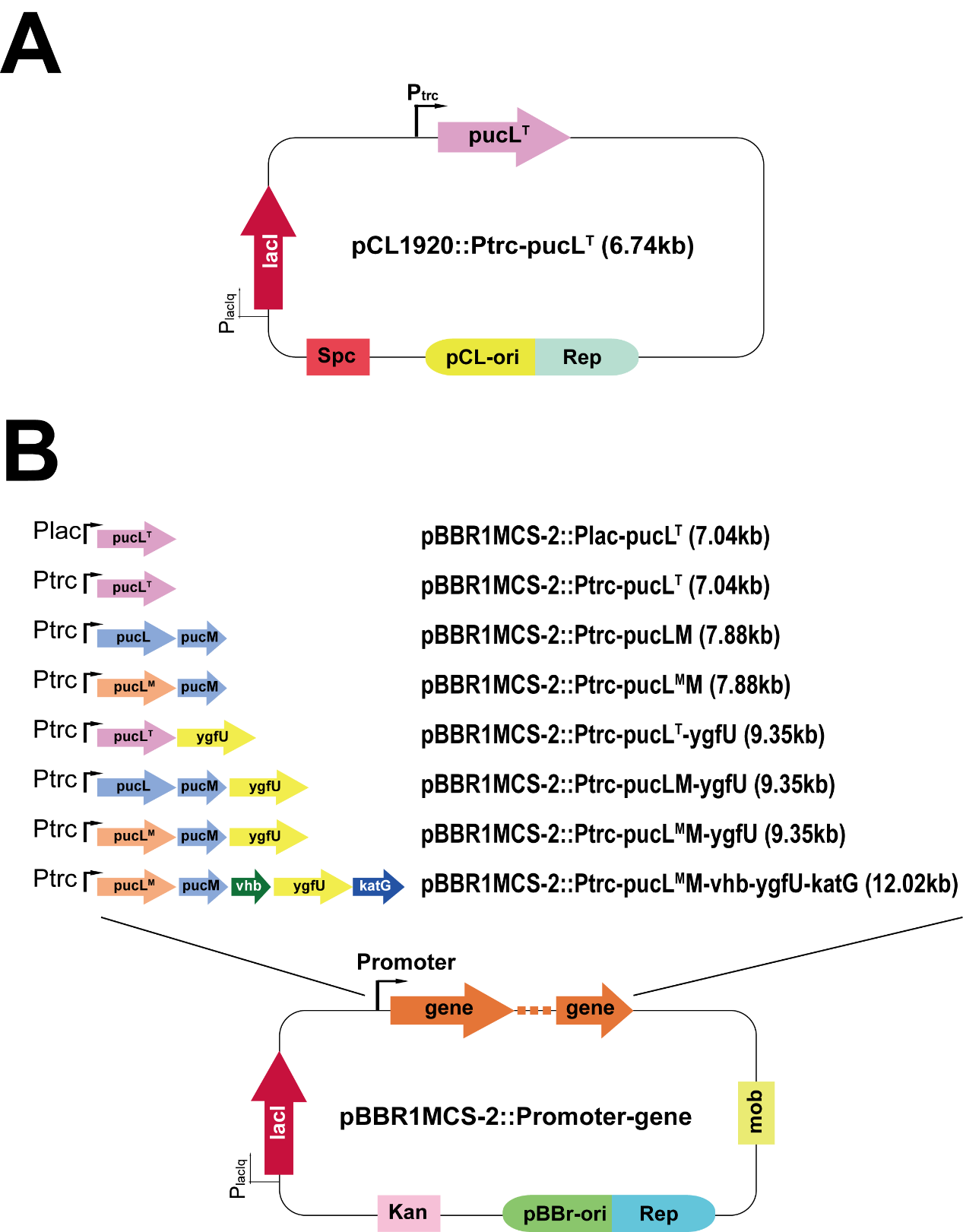


**Supplementary Figure S6. The schematic diagram of the plasmids used in this study.**

**References**

1. Kovach ME, Elzer PH, Hill DS, Robertson GT, Farris MA, Roop RM, et al. 4 New Derivatives of the Broad-Host-Range Cloning Vector Pbbr1mcs, Carrying Different Antibiotic-Resistance Cassettes. Gene 1995; 166:175-176.

2. Lerner CG, Inouye M. Low Copy Number Plasmids for Regulated Low-Level Expression of Cloned Genes in Escherichia-Coli with Blue White Insert Screening Capability. Nucleic Acids Res 1990; 18:4631-4631.

3. Amann E, Ochs B, Abel KJ. Tightly Regulated Tac Promoter Vectors Useful for the Expression of Unfused and Fused Proteins in Escherichia-Coli. Gene 1988; 69:301-315.
